# Soil water potential and temperature sum during reproductive growth control seed dormancy in *Alopecurus myosuroides* Huds.

**DOI:** 10.1002/ece3.4249

**Published:** 2018-06-12

**Authors:** Alexander Menegat, Per Milberg, Anders T. S. Nilsson, Lars Andersson, Giulia Vico

**Affiliations:** ^1^ Department of Crop Production Ecology Swedish University of Agricultural Sciences (SLU) Uppsala Sweden; ^2^ Department of Physics, Chemistry and Biology (IFM) Linköping University Linköping Sweden; ^3^ Department for Biosystems and Technology Swedish University of Agricultural Sciences (SLU) Alnarp Sweden

**Keywords:** black‐grass, generative growth phase, integrated weed management, soil seed bank dynamics, soil water availability

## Abstract

The sustainable management of unwanted vegetation in agricultural fields through integrated weed control strategies requires detailed knowledge about the maternal formation of primary seed dormancy, to support the prediction of seedling emergence dynamics. This knowledge is decisive for the timing of crop sowing and nonchemical weed control measures. Studies in controlled environments have already demonstrated that thermal conditions and, to some extent, water availability during seed set and maturation has an impact on the level of dormancy. However, it is still unclear if this applies also under field conditions, where environmental stressors and their timing are more variable. We address this question for *Alopecurus myosuroides* in south‐western Sweden. We quantified the effects of cumulated temperature and precipitation as well as soil water potential during the reproductive growth phase of *A myosuroides* on primary seed dormancy under field conditions. Empirical models differing in focal time intervals and, in case of soil water potential, focal soil depths were compared regarding their predictive power. The highest predictive power for the level of primary dormancy of *A. myosuroides* seeds was found for a two‐factorial linear model containing air temperature sum between 0 and 7 days before peak seed shedding as well as the number of days with soil water potential below field capacity between 7 and 35 days before peak seed shedding. For soil water potential, it was found that only the top 10 cm soil layer is of relevance, which is in line with the shallow root architecture of *A. myosuroides*. We conclude that for this species the level of dormancy depends on the magnitude and timing of temperature and water availability during the reproductive growth phase. Water availability appears to be more important during maternal environmental perception and temperature during zygotic environmental perception.

## 1 INTRODUCTION

*Alopecurus myosuroides* Huds. (black‐grass), an annual grass species native to Eurasia, is one of the main weed species in winter‐sown crops in the temperate regions of Europe. This species has drawn major attention of farmers, advisors, and researchers since the beginning of herbicide‐dominated agricultural practice in central Europe. Its low germination base temperature in combination with short primary dormancy is allowing *A. myosuroides* a wide germination period, ranging from late summer into early winter, when the competitive abilities of winter annual crops are low. In addition, *A. myosuroides* is able to complete its life cycle well before crop harvest. Hence, this species is very well adapted to agricultural cropping systems based on winter annual crops and in particular to reduced soil tillage systems, as it mainly germinates from soil depths of <5 cm.

Despite substantial research efforts, *A. myosuroides* is still one of the most difficult to control weed species (Lutman, Moss, Cook, & Welham, 2013; Maréchal, Henriet, Vancutsem, & Bodson, 2012). The increasing number of herbicide resistance cases suggests that no single control measure is likely to succeed in the long‐term, rather than integrated weed management strategies are required. Integrated weed management strategies (IWM) aim to combine the use of agronomic, mechanical and chemical measures for prevention and control of weeds. Knowledge about the dormancy status could support decision making in IWM regarding type of soil cultivation, timing of crop sowing and choice of pre‐ and post‐sowing weed control measures. For *A. myosuroides*, the main germination and emergence time falls into the period of winter annual crop sowing in early autumn. Weed plants emerging before crop sowing can be easily removed through soil cultivation, whereas plants emerging after crop sowing have to be controlled mechanically or by the use of selective herbicides. The aim of an IWM approach is thus to minimize the proportion of weed seeds germinating and emerging after crop sowing, when nonchemical control options are least applicable and effective. However, due to their multifactorial nature, IWM strategies are difficult to evaluate through field trials and therefore modelling approaches are strongly required (Bastiaans, Kropff, Goudriaan, & Laar, 2000). Seed dormancy and dormancy release play a key role in population dynamics models, as they have direct bearing on the species’ ability to accumulate in the soil seed bank as well as on the timing and amount of seedling emergence.

In general, a viable seed is considered as dormant if it does not have the capacity to germinate within a specified period of time and under any combination of environmental factors that are otherwise favorable for its germination (Baskin & Baskin, 2004). As long as the physiological background of seed dormancy is not completely understood, a more clear definition is rather difficult as dormancy can basically only be measured by the absence of germination (Finch‐Savage & Leubner‐Metzger, 2006). *Alopecurus myosuroides* generally shows a very short primary dormancy, so that the majority of seeds germinate within the first year after seed shedding (Brenchley & Warington, 1930). Previous studies showed that the level of dormancy between seed batches collected around similar dates within a region may vary greatly (Andersson & Milberg, 1998). These effects are likely to be of phenotypic nature and driven by small differences in maternal conditions during seed set and maturation (Andersson & Espeby, 2009; Andersson & Milberg, 1998; Swain, Hughes, Cook, & Moss, 2006).

A study by Swain et al. (2006) is suggesting the hypothesis, that the level of primary dormancy in *A. myosuroides* seeds might be associated with air temperature conditions and, to some extent, with water availability during seed ripening. The study is based on earlier investigations, where similar effects could describe dormancy in, for example, *Avena fatua* and *Cenchrus ciliaris* (Sexsmith, 1969; Sharif‐Zadeh & Murdoch, 2000). The mentioned studies were mostly conducted under controlled conditions, thus neglecting the effect of environmental stochasticity as well as intra‐ and interspecific competition. The effect of timing of temperature and water stress during seed production and seed ripening as well as the interaction between both factors are still poorly understood. Regarding the prediction of the level of seed dormancy, it is further unclear whether precipitation or the actual soil water potential is the more meaningful model variable.

In the present contribution, we quantitatively assess the importance of temperature, precipitation and soil water potential during the maternal reproductive growth phase for the primary dormancy of *A. myosuroides* seeds. For each of these aspects and their combinations, we study critical time periods and, in the case of soil water potential, critical soil layers. We combine seed dormancy data with freely available soil and meteorological data towards a site‐specific seed dormancy model. The suggested model can support the development of population dynamics models and decision support systems for weed control.

## 2 METHODS

### 2.1 Species biology, abundance and relevance in Sweden

**Figure 1:**
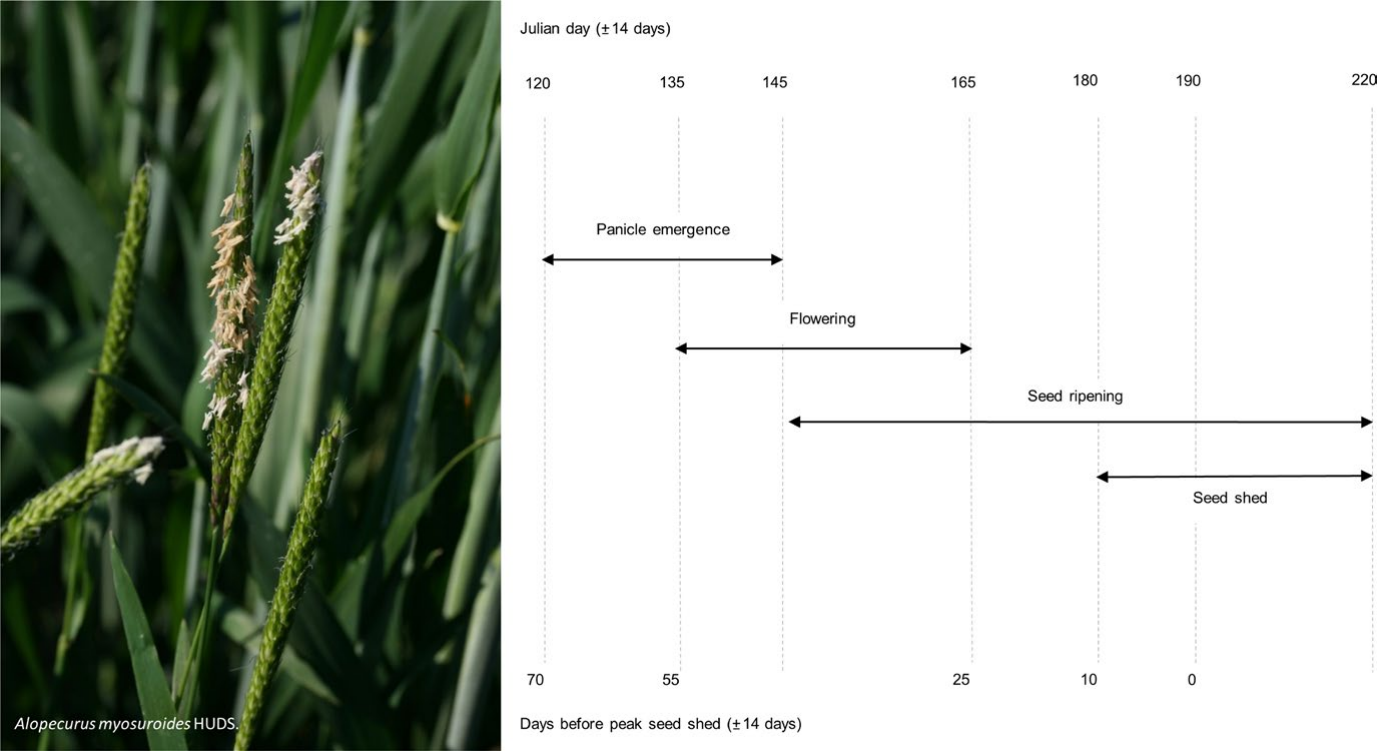
Left: panicles and inflorescence of *A. myosuroides* Huds. Right: the typical timing of *A. myosuroides* reproduction in Southern Sweden

**Figure 2:**
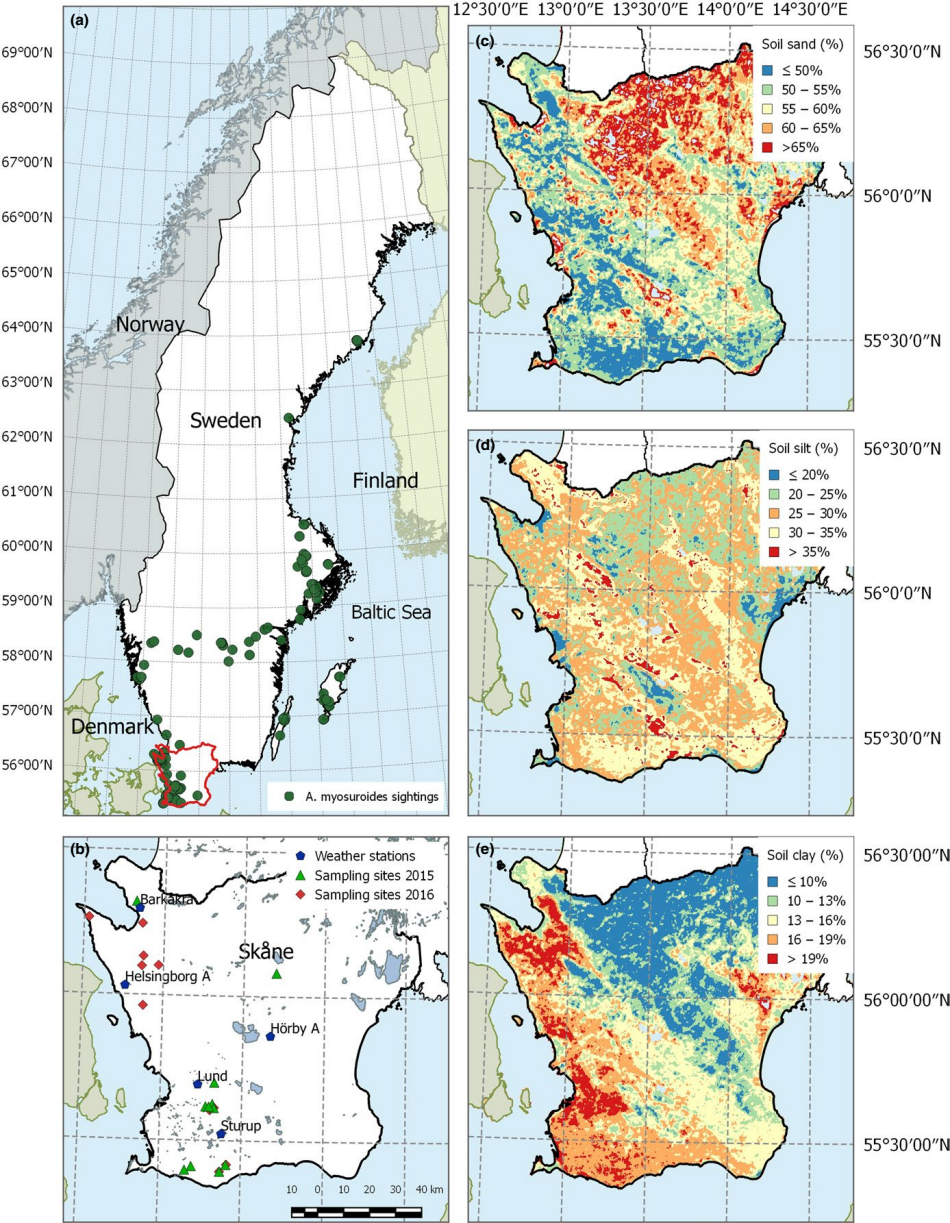
(a) *A. myosuroides* distribution in Sweden (ArtDatabanken, SLU 2016). (b) The study region Skåne with sampling sites in 2015 (triangles) and 2016 (diamonds) and weather stations (dots). (c–e) sand, silt, and clay content in Skåne, 1 km × 1 km grid (ISRIC 2016)

*Alopecurus myosuroides* Huds. (Figure 1, left) is one of the main weed species in winter‐sown crops in the temperate regions of Europe. The species is most abundant on heavy soils of poor structure and drainage (Barallis, 1968). In Sweden, *A. myosuroides* can be found along the coastal regions up to 64°N but its importance as an agricultural weed is currently limited to the temperate winter cereal growing regions below 60°N (Figure 2a).

*Alopecurus myosuroides* mainly germinates between September and December from soil depths of maximum 5 cm. It overwinters in a two‐leaf to five‐tiller stage with vigorous resumption of growth in spring. The germination base temperature is estimated to be at around 0°C (Colbach, Chauvel, Dürr, & Richard, 2002). Plants have a shallow root system and reach up to 80 cm in height (Naylor, 1972). *Alopecurus myosuroides* is allogamous and self‐incompatible with a high level of genetic polymorphism within populations and low genetic diversity between populations (Chauvel & Gasquez, 1994). Autumn germinated plants start flowering in mid of May the earliest with anthesis beginning at the apex of the panicle and continuing toward the base of the panicle within 7–10 days (Holm, Doll, Holm, Pancho, & Herberger, 1997). The protogynous flowers are wind‐pollinated (Naylor, 1972). Seed shedding starts at the end of June and can last until mid of August (Figure 1, right). Although the dispersal unit of *A. myosuroides* is a spikelet, the term ‘seed’ is used for consistency with other publications.

### 2.2 Study site description

*Alopecurus myosuroides* seed samples were collected in the year 2015 and 2016 from in total 23 agricultural fields along the west coastal region of Skåne, south‐western Sweden (Figure 2a,b). The dominant crops in the sampling region are autumn‐sown cereals, grown over half of the arable land and typically in rotation with winter oilseed rape (Statistics Sweden, Agricultural statistics 2016). The climate is characterized as warm and temperate oceanic (Köppen‐Geiger climate classification: Cfb). Annual average temperature in the region ranges between 7 and 10°C with annual rainfalls increasing from 650 mm inland to 1,100 mm towards the coast. The year 2015 was similar to the long‐term rainfall and temperature average. In contrast, 2016 was comparatively dry especially during the reproductive growth phase of *A. myosuroides* in May and June (Supporting information Figures S1 and S2).

The selected sampling sites are covering the typical soil textures of the study region; clay loam (ClLo), sandy clay loam (SaClLo) and sandy loam (SaLo). Soil sand content ranged between 36% and 60%, soil silt content between 21% and 36% and soil clay content between 14% and 31%. For each sampling site, we selected the closest weather station within the SMHI (Swedish Meteorological and Hydrological Institute) network as representative for the weather conditions at the sampling site. Distances to the closest weather station ranged between 3 and 24 km (Supporting information Table S1).

All 23 sampling sites have a similar agronomic history, with winter wheat grown as the main crop for two consecutive years and in rotation with one year of winter oilseed rape or barley. Fertilizer and herbicides were used in accordance to the common farming practice of the region. Herbicide choice during the past decade was similar at all sampling sites and dominated by compounds belonging to the acetohydroxyacid synthesis inhibitors (ACCase‐inhibitors) or fatty acid synthesis inhibitors (ALS‐inhibitors). Due to the similar agronomic history, we can assume a similar selection pressure for all sampled *A. myosuroides* populations. Winter wheat was grown at all sampling sites in the respective year of seed collection.

### 2.3 Seed sampling and dormancy determination

*Alopecurus myosuroides* seeds were collected after 10%–30% of the seeds had been shed (subsequently referred to as peak seed shedding). Seed samples were obtained by gently shaking minimum 200 panicles per sampling site (one panicle per plant) into a paper bag. Panicles were selected randomly within each sampling site through crossing the infested areas of the fields in a w‐manner. Sampled fields had a minimum size of 10 ha. Samples were cleaned and spikelets without caryopsis were removed by hand. Previous studies showed that the proportion of viable seeds is equal to the percentage of spikelets with caryopsis (Moss, 1983), why we assume that all remaining seeds were viable. All dormancy tests were carried out within 3–7 days after seed sampling. Meanwhile, seed samples were stored under dry conditions at 20°C. Storage under dry conditions does not significantly influence the dormancy status of the seeds (Andersson & Espeby, 2009). Germination rate was determined by placing 50 seeds into 9 cm Petri dishes containing three layers of filter paper (Whatman Number 1) and 7 ml 0.2% potassium nitrate solution (2 g KNO_3_/L in deionized water). As shown in previous studies, the use of potassium nitrate has only marginal effect on breaking the dormancy of *A. myosuroides* seeds (Swain et al., 2006). Each sample was replicated six times. Petri dishes were placed in an incubator set to 17°C during daytime (14 hr) and 11°C during night (10 hr). The selected temperatures are representative for the average temperatures in southern Sweden during the autumn germination period. After 7, 11, 14, 17, and 21 days, the accumulated number of germinated seeds per Petri‐dish were counted. For consistency with other publications, the number of germinated seeds after 14 days was used as a measure of the proportion of nondormant seeds (Swain et al., 2006).

### 2.4 Explanatory variables

As potential explanatory variables for the observed seed dormancy, we considered air temperature sums and precipitation sums as suggested by previous studies (Swain et al., 2006). In addition, we considered the duration of low soil water potential. Temperature sum was calculated as

(1) $$\Sigma T_{i,j}=\sum_{i}^{j}\frac{T_{\max}-T_{\min}\;}{2}$$

where $T_{\max}$ is the maximum daily temperature (°C), $T_{\min}$ is the minimum daily temperature (°C), *i* and *j* refer to the first and last days of the period considered. The minimum duration of a time period was 7 days and ranged between zero and 56 days before seed shedding, whereby 56 days before seed shedding marks the beginning of flowering (Figure 1 right). When considering a germination base temperature of 0°C, Equation (1) equals growing degree days (GDD).

The precipitation sum was determined as

(2) $$\Sigma P_{i,j}=\sum_{i}^{j}P$$

where P is the daily total precipitation (mm). The temperature and precipitation sums were tested as explanatory variables separately as well as in all possible combinations and for various time periods. The third tested set of models was based on soil water potential. The number of days where the daily average soil water potential was below a certain threshold were counted for similar time periods as described above.

(3) $$\Sigma \psi_{i,j,k}=\sum_{i}^{j}\;\text{days with}\;\left\langle \psi \right\rangle <\left\langle \psi_{k}\right\rangle$$

where ⟨ψ⟩ is the average soil water potential over the day and $\left\langle \psi_{k}\right\rangle$ is the soil water potential threshold tested. The tested soil water potential thresholds ($\left\langle \psi_{k}\right\rangle$) ranged from −0.033 MPa (field capacity) to −1.5 MPa (permanent wilting point) in −0.1 MPa steps. The soil water potential thresholds were tested for three different soil layers; 0–10 cm, 10–20 cm, and 20–30 cm soil depth.

Soil water potential was tested separately for its ability to describe the observed seed dormancy data as well as in all possible combinations with temperature sum. To avoid multicollinearity, models containing soil water potential and precipitation sum were not considered.

### 2.5 Soil moisture model

As no soil water potential data were available for the sampling sites and years, a soil moisture model was used to estimate soil water potential over 1‐cm soil layers, from the surface to 0.3 m depth (Spokas & Forcella, 2009). The utilized model estimates soil moisture based on soil features as well as based on daily minimum and maximum temperature and precipitation. The model has been validated for numerous soil and climate conditions, including locations where snow plays a role (Perreault, Chokmani, Nolin, & Bourgeois, 2013; Spokas & Forcella, 2009).

The required soil feature data (sand, silt, clay and organic matter content) were not directly available for all sampling sites. Therefore the required site‐specific estimates were derived from ISRIC—World Soil Information (ISRIC 2016). The resulting detailed model parametrization is given in Supporting information Tables S1 and S2.

The model was validated against soil moisture data measured in 2015 and 2016 in Hyltemossa, Skåne County, Sweden (ICOS 2016). Hyltemossa marks the center of our study region, with comparable soil and climate conditions. Soil moisture was measured in three different soil depths: 0–6 cm as well as at 10 cm and 30 cm. Model performance was assessed along the root mean square error (RMSE) and bias. In addition, the *d* index of agreement was calculated as

(4) $$d=1-\frac{\sum_{i=1}^{N}{\left(O_{i}-E_{i}\right)}^{2}}{\sum_{i=1}^{N}{\left(\left|O_{i}-\overset{¯}{O}\right|+\left|E_{i}-\overset{¯}{O}\right|\right)}^{2}}$$

where $E_{i}$ denotes estimated values, $O_{i}$ denotes observed values and *N* is the number of observations (Willmott, 1981). The *d* index is unit‐less and varying between 0 and 1, whereby 1 is indicating total agreement between estimated and observed values. The validation results can be found in the supporting material (Figure S3 and S4 and Table S3).

### 2.6 Model selection

The Akaike Information Criterion (AIC) was used for model comparison and model selection purposes (Akaike, 1973). Due to the small sample sizes, the second‐order Akaike Information Criterion, AIC_c_, with bias correction term was used according to Equation (5):

(5) $${\text{AIC}}_{c}=-2\ln\left(\text{likelihood}\right)+2p+\frac{2p\left(p+1\right)}{n-p-1}$$

where *n* denotes the sample size and *p* denotes the number of parameters in the model.

The best models were compared by means of Akaike weights (*w*_*i*_), representing the relative weight of evidence for model *i* (Johnson & Omland, 2004).

(6) $$w_{i}=\frac{\exp\left(-0.5\Delta_{i}\right)}{\sum_{r=1}^{N}\exp\left(-0.5\Delta_{r}\right)}$$

where

(7) $$\Delta_{i}={\text{AIC}}_{i}-{\text{AIC}}_{\min}$$

and AIC_i_ denotes the AIC_c_ in comparison and AIC_min_ denotes the lowest AIC_c_ in comparison (‘best’ model AIC_c_).

## 3 RESULTS

### 3.1 Observed climatic and hydrologic conditions

The average temperature sum during the generative growth phase of *A. myosuroides* accumulated to 911°C in 2016, that is, 78°C higher than 2015. The number of days with an average soil water potential below −0.033 MPa (field capacity) was 11 days larger in 2016 compared to 2015. The number of days with an average soil water potential below −1.5 MPa (permanent wilting point) was 16 days larger in 2016 compared to 2015. The average proportion of dormant seeds in 2016 was 13% lower than in 2015 (Table 1).

**Table 1:** Sampling year comparison

Year	Proportion of dormant seeds (%)		10%–30% seed shed reached (day)		Precipitation sum during generative growth phase (mm)^a^		Mean temperature during generative growth phase (°C)^a^		Temperature sum during generative growth phase (°C)^a^		Days with mean ψ < FC during generative phase (2–10 cm soil depth)^a^		Days with mean ψ < PWP during generative phase (2–10 cm soil depth)^a^	
2015	68		199		101		15		834		30		9	
2016	55	(−13%)	189	(−10 days)	111	(+10 mm)	16	(+1°C)	911	(+77°C)	41	(+11 days)	25	(+16 days)

*Notes*

Precipitation, temperature and SMP related values are weighed by the number of sampling sites associated per weather station. FC: field capacity, *−*0.033 MPa; PWP: permanent wilting point, *−*1.5 MPa. Differences between 2015 and 2016 are given in brackets.

a *−*56 to 0 days before seed shedding (10%–30% seed shed).

### 3.2 Model selection

**Figure 3:**
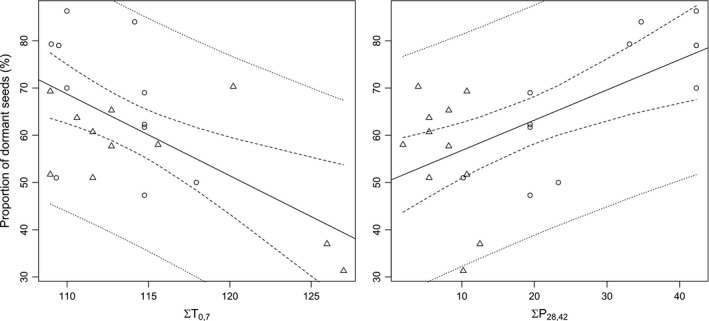
Left: linear relationship between proportion of dormant seeds (%) and temperature sum 0–7 days before seed shedding (adjusted R^2^ = 0.345, y=259.04−1.73ΣT). Right: linear relationship between proportion of dormant seeds (%) and precipitation sum (mm) 28–42 days before seed shedding (adjusted R^2^=0.359, y=50.38+0.64ΣP). Dashed line marks the 95% confidence interval for the regression, the dotted line marks the 95% prediction interval

The second‐order Akaike Information Criterion (AIC_c_) for the explanatory variable temperature sum varied in dependency of the considered time period, although the AIC_c_ over time did not follow a clear pattern (Supporting information Table S4). The highest correlation between temperature sum and seed dormancy was found for the time frame 0–7 days before seed shedding with an adjusted *R* ^2^ of 0.345 (Figure 3, left).

The correlation between seed dormancy and precipitation sum was only significant between 28 and 42 days before seed shedding, with an adjusted *R* ^2^ of 0.359 (Figure 3 right).

**Figure 4:**
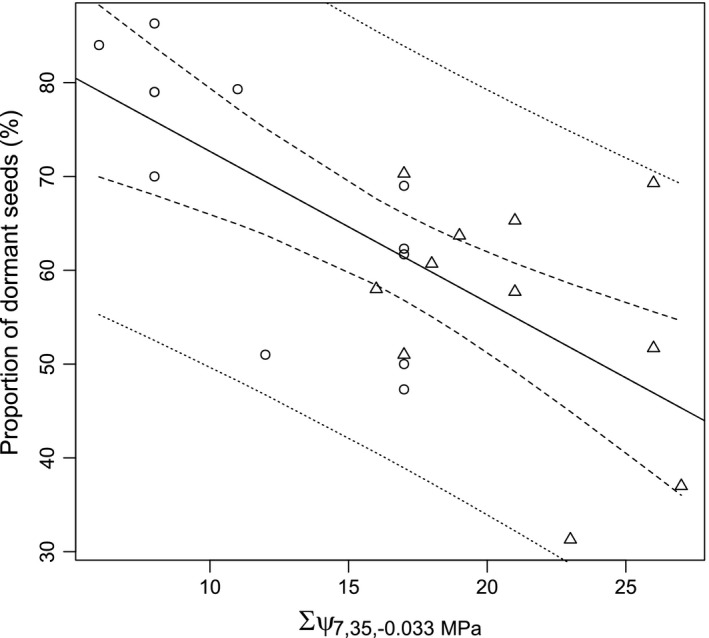
Linear relationship between proportion of dormant seeds and the number of days, between 7 and 35 days before shedding, with mean soil water potential <−0.033 MPa (field capacity, FC). Dashed line marks the 95% confidence interval for the regression, the dotted line marks the 95% prediction interval. Adjusted R^2^ = 0.454. Regression equation: y* *=* *88.76* − *1.61*ψ*_7,35, *−*0.033 MPa_

According to the AIC_c_ comparisons, the number of days with mean soil water potential below field capacity (<−0.033 MPa) was found as the most relevant soil water potential threshold. The AIC_c_ values appeared to be lowest for the observation period between 7 and 35 days before seed shedding (Figure 4; Supporting information Table S5) and considering the topsoil layer (2–10 cm soil depth).

In a second step, all possible models combining temperature sum and soil water potential were compared, considering the complete set of soil layers, time frames, and soil water potential thresholds. Models including soil water potential and precipitation sum had to be omitted due to multicollinearity.

The two parameter linear model considering temperature sum for the time period 0–7 days before seed shedding and number of days with soil water potential below field capacity for the time period 7–35 days before seed shedding appeared to be the most promising model due to the lowest AIC_c_ value (Table**** 2). An adjusted *R* ^2^ of 0.566 was obtained for this model. A probability of 82% (*w*_*i*_ = 0.819) was calculated that this model is the best model among all the tested ones.

**Table 2:** Best model comparison

Model	AIC_c_	ΔAIC_c_	*w*_*i*_	Adjusted *R* ^2^
Σ*T*_0,7_	183.14	7.2	0.022	0.345
Σ*P*_28,42_	182.63	6.7	0.029	0.359
Σψ_7,35,−0.033_	179.63	3.7	0.130	0.454
Σ*T*_0,7_ + Σψ_7,35,−0.033_	175.94	0.0	0.819	0.566

**Figure 5:**
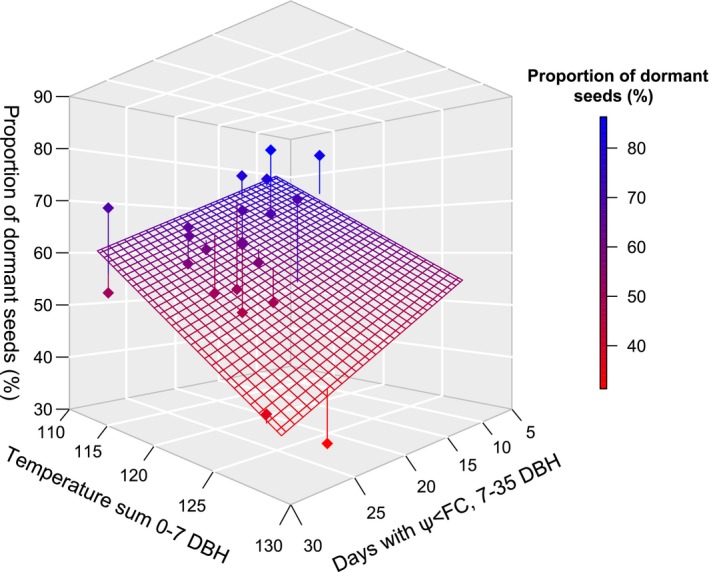
Two parameter linear relationship between proportion of dormant seeds (*y*) and temperature sum 0–7 days before shedding (×1) and the number of days, between 7 and 35 days before shedding, with mean soil water potential <−0.033 MPa (×2). Model adjusted *R* ^2^ = 0.566. Regression equation $y=208.35-1.10\Sigma T_{0,7}-1.23\psi_{7,35,-0.033\;\mathrm{MPa}}$

Focusing on the variables with the highest explanatory power, the estimated proportion of dormant seeds is summarized in Figure 5. The proportion of dormant seeds was found to be lowest when temperature sum during seed ripening (zygotic environment) was high in combination with a high number of days with average soil moisture below field capacity during flowering and seed set (maternal environment).

## 4 DISCUSSION

The aim of this study was to describe the effect of temperature, precipitation, and soil water availability, during the maternal reproductive growth phase, on the primary dormancy of *A. myosuroides* seeds. For the mentioned explanatory variables we studied different critical time periods as well as, in the case of soil water availability, different soil layers. The studied time periods covered the maternal perception during panicle emergence and flowering as well as the zygotic perception during seed ripening and seed shedding. Furthermore, we demonstrate the feasibility of using standard, freely available, soil and meteorological data for modelling the level of primary dormancy in *A. myosuroides* seeds.

We could confirm under field conditions the hypothesis that temperature and water availability for the maternal plant have a pronounced impact on seed dormancy. Beyond that, the results could show that the timing of specific levels of temperature and water availability is decisive for the level of dormancy. For the tested populations, the critical period for temperature effects appeared to be during the seed ripening phase (0–7 days before peak seed shedding). In contrast, the critical period for water availability was found during the flowering and early seed ripening period (7–35 days before peak seed shedding). These results indicate that dormancy in *A. myosuroides* is a response to both the maternal and zygotic environment. Water availability appears to be more important during maternal environmental perception and temperature during zygotic environmental perception. The maternal perception of water deficit could potentially cause the modification of seed‐coat tissues or the inheritance of maternal epigenetic states (Penfield & MacGregor, 2017). As summarized by Kendall and Penfield (2012) and Penfield and MacGregor (2017) the zygotic perception of temperature has a strong effect on gene transcript levels and gene expression controlling the abscisic acid—gibberellic acid balance of seeds.

The variation in dormancy, so‐called bet hedging, documented here and elsewhere (Andersson & Espeby, 2009; Carta, Probert, Puglia, Peruzzi, & Bedini, 2016; Milberg & Andersson, 1998; Milberg, Andersson, & Noronha, 1996), is beside rapid evolution and adaptive plasticity a third way for adapting to variable environments (Seger & Brockmann, 1987). Dormant seeds build the population hedge against the risk of reproductive failure (Gremer & Venable, 2014). As shown by Gremer and Venable (2014), bet hedging is crucial for population survival in the notoriously unpredictable environment on arable land and at the same time, it creates obstacles for predicting the timing of seedling emergence. The observed phenotypic variation has an evolutionary advantage as it ensures that germination is not exclusively confined to one short time period in a single year (Fraser & Kærn, 2009; Rubio de Casas, Donohue, Venable, & Cheptou, 2015). Unlike in summer annual species, cold stratification of seeds does not seem to level out primary dormancy in autumn‐germinating species like *A. myosuroides* (Milberg & Andersson, 1998).

In conclusion, the results showed which environmental variables are key to explain primary seed dormancy in *A. myosuroides*. The presented dormancy model could enable site‐specific dormancy forecast with a minimum of field observation and freely available soil and weather data. Beneath the use of this model in practical agriculture for the planning of crop sowing and weed control measures, it can be used for basic population dynamics research. Of particular interest would be the effect of climate change on seed dormancy and hence on prospective population growth rates.

## CONFLICT OF INTEREST

None declared.

## AUTHOR CONTRIBUTIONS

All authors conceived the ideas and designed methodology; A.N. and L.A. collected the data; A.M. analyzed the data and model results with the support of G.V.; A.M. and G.V. led the writing of the manuscript. P.M. and A.M. led and wrote the discussion of the results. All authors contributed critically to the drafts and gave final approval for publication.

## References

[ece34249-bib-0001] Akaike, H. (1973). Information theory and an extension of the maximum likelihood principle In Petrov B. N., & Caski F. (Eds.), Proceedings of the second international symposium on information theory (pp. 267–281). Budapest, Hungary: Akademiai Kiado.

[ece34249-bib-0002] Andersson, L. , & Espeby, L. A. (2009). Variation in seed dormancy and light sensitivity in *Alopecurus myosuroides* and *Apera spica‐venti* . Weed Research, 49, 261–270. 10.1111/j.1365-3180.2009.00695.x

[ece34249-bib-0003] Andersson, L. , & Milberg, P. (1998). Variation in seed dormancy among mother plants, populations and years of seed collection. Seed Science Research, 8(1), 29–38. 10.1017/S0960258500003883

[ece34249-bib-0004] Barallis, G. (1968) Ecology of blackgrass, *9th British Weed Control Conference*, pp. 6–8.

[ece34249-bib-0005] Baskin, J. M. , & Baskin, C. C. (2004). A classification system for seed dormancy. Seed Science Research, 14, 1–16.

[ece34249-bib-0006] Bastiaans, L. , Kropff, M. J. , Goudriaan, J. , & Laar, H. H. Van (2000). Design of weed management systems with a reduced reliance on herbicides poses new challenges and prerequisites for modeling crop ‐ weed interactions. Field Crops Research, 67, 161–179. 10.1016/S0378-4290(00)00091-5

[ece34249-bib-0007] Brenchley, W. E. , & Warington, K. (1930). The weed seed population of arable soil. I. Numerical estimation of viable seeds and observations on their natural dormancy. Journal of Ecology, 18, 235–272. 10.2307/2256005

[ece34249-bib-0008] Carta, A. , Probert, R. , Puglia, G. , Peruzzi, L. , & Bedini, G. (2016). Local climate explains degree of seed dormancy in *Hypericum elodes* L. (Hypericaceae). Plant Biology, 18, 76–82. 10.1111/plb.12310 25662792

[ece34249-bib-0009] Chauvel, B. , & Gasquez, J. (1994). Relationships between genetic polymorphism and herbicide resistance within *Alopecurus myosuroides* Huds. Heredity, 72, 336–344. 10.1038/hdy.1994.50

[ece34249-bib-0010] Colbach, N. , Chauvel, B. , Dürr, C. , & Richard, G. (2002). Effect of environmental conditions on *Alopecurus myosuroides* germination. I. Effect of temperature and light. Weed Research, 42, 210–221. 10.1046/j.0043-1737.2002.00279.x

[ece34249-bib-0011] Finch‐Savage, W. E. , & Leubner‐Metzger, G. (2006). Seed dormancy and the control of germination. New Phytologist, 171, 501–523. 10.1111/j.1469-8137.2006.01787.x 16866955

[ece34249-bib-0012] Fraser, D. , & Kærn, M. (2009). A chance at survival: Gene expression noise and phenotypic diversification strategies. Molecular Microbiology, 71, 1333–1340. 10.1111/j.1365-2958.2009.06605.x 19220745

[ece34249-bib-0013] Gremer, J. R. , & Venable, D. L. (2014). Bet hedging in desert winter annual plants: Optimal germination strategies in a variable environment. Ecology Letters, 17, 380–387. 10.1111/ele.12241 24393387

[ece34249-bib-0014] Holm, L. , Doll, J. , Holm, E. , Pancho, J. V. , & Herberger, J. P. (1997). World weeds: Natural histories and distribution. New York, NY: Wiley.

[ece34249-bib-0015] ICOS . (2016). ICOS ‐ Integrated Carbon Observation System, http://www.icos-sweden.se

[ece34249-bib-0016] ISRIC . (2016). ISRIC ‐ WDC Soils, http://www.soilgrids.org

[ece34249-bib-0017] Johnson, J. B. , & Omland, K. S. (2004). Model selection in ecology and evolution. Trends in Ecology and Evolution, 19, 101–108. 10.1016/j.tree.2003.10.013 16701236

[ece34249-bib-0018] Kendall, S. , & Penfield, S. (2012). Maternal and zygotic temperature signalling in the control of seed dormancy and germination. Seed Science Research, 22, S23–S29. 10.1017/S0960258511000390

[ece34249-bib-0019] Lutman, P. J. W. , Moss, S. R. , Cook, S. , & Welham, S. J. (2013). A review of the effects of crop agronomy on the management of *Alopecurus myosuroides* . Weed Research, 53, 299–313. 10.1111/wre.12024

[ece34249-bib-0020] Maréchal, P.‐Y. , Henriet, F. , Vancutsem, F. , & Bodson, B. (2012). Ecological review of black‐grass (*Alopecurus myosuroides* Huds.) propagation abilities in relationship with herbicide resistance. Biotechnologie, Agronomie, Société et Environnement, 16, 103–113.

[ece34249-bib-0021] Milberg, P. , & Andersson, L. (1998). Does cold stratification level out differences in seed germinability between populations? Plant Ecology, 134, 225–234. 10.1023/A:1009793119466

[ece34249-bib-0022] Milberg, P. , Andersson, L. , & Noronha, A. (1996). Seed germination after short‐duration light exposure: Implications for the photo‐control of weeds. Journal of Applied Ecology, 33, 1469–1478. 10.2307/2404785

[ece34249-bib-0023] Moss, S. R. (1983). The production and shedding of *Alopecurus myosuroides* Huds. seeds in winter cereals crops. Weed Research, 23, 45–51. 10.1111/j.1365-3180.1983.tb00519.x

[ece34249-bib-0024] Naylor, R. E. L. (1972). *Alopecurus Myosuroides* Huds. (*A. Agrestis* L.). Journal of Ecology, 60, 611–622. 10.2307/2258364

[ece34249-bib-0025] Penfield, S. , & MacGregor, D. R. (2017). Effects of environmental variation during seed production on seed dormancy and germination. Journal of Experimental Botany, 68, 819–825. 27940467 10.1093/jxb/erw436

[ece34249-bib-0026] Perreault, S. , Chokmani, K. , Nolin, M. C. , & Bourgeois, G. (2013). Validation of a soil temperature and moisture model in Southern Quebec, Canada. Soil Science Society of America Journal, 77, 606–617. 10.2136/sssaj2012.0311

[ece34249-bib-0027] Rubio de Casas, R. , Donohue, K. , Venable, D. L. , & Cheptou, P. O. (2015). Gene‐flow through space and time: Dispersal, dormancy and adaptation to changing environments. Evolutionary Ecology, 29, 813–831. 10.1007/s10682-015-9791-6

[ece34249-bib-0028] Seger, J. , & Brockmann, H. J. (1987). What is bet‐hedging? In Harvey P. H., & Partridge L. (Eds.), Oxford surveys in evolutionary biology, Vol. 4 (pp. 182–211). Oxford, UK: Oxford University Press.

[ece34249-bib-0029] Sexsmith, J. J. (1969). Dormancy of wild oat seed produced under various temperature and moisture conditions. Weed Science, 17, 405–407.

[ece34249-bib-0030] Sharif‐Zadeh, F. , & Murdoch, A. J. (2000). The effects of different maturation conditions on seed dormancy and germination of *Cenchrus ciliaris* . Seed Science Research, 10, 447–457. 10.1017/S0960258500000490

[ece34249-bib-0031] Spokas, K. , & Forcella, F. (2009). Software tools for weed seed germination modeling. Weed Science, 57, 216–227. 10.1614/WS-08-142.1

[ece34249-bib-0032] Statistics Sweden . (2016). Agricultural statistics. Sweden: Örebro.

[ece34249-bib-0033] Swain, A. J. , Hughes, Z. S. , Cook, S. K. , & Moss, S. R. (2006). Quantifying the dormancy of *Alopecurus myosuroides* seeds produced by plants exposed to different soil moisture and temperature regimes. Weed Research, 46, 470–479. 10.1111/j.1365-3180.2006.00532.x

[ece34249-bib-0034] Willmott, C. J. (1981). On the validation of models. Physical Geography, 2, 184–194.

