# Supplementary material for: Soil water potential and temperature sum during reproductive growth control seed dormancy in Alopecurus myosuroides Huds
Source: Ecol Evol. 2018 Jun 12;8(14):7186–94. doi: 10.1002/ece3.4249 (PMC6065331; doi:10.1002/ece3.4249)
Supplement: Supplementary file 1 [file ECE3-8-7186-s001.docx]

**Soil water potential and temperature sum during reproductive growth controls seed dormancy in *Alopecurus myosuroides* Huds.**

**Alexander Menegat^1^*, Per Milberg^2^, Anders T. S. Nilsson^3^, Lars Andersson^1^ and Giulia Vico^1^**

*^1^Department of Crop Production Ecology, Swedish University of Agricultural Sciences (SLU), Uppsala, Sweden*

*^2^Department of Physics, Chemistry and Biology (IFM), Linköping University, Sweden*

*^3^Institute for Biosystems and Technology, Swedish University of Agricultural Sciences (SLU), Alnarp, Sweden*

*Correspondence author. E-mail: alexander.menegat@slu.se

**Supporting information**

## SOIL MOISTURE MODEL EVALUATION

Soil moisture data for evaluation of the performance of the soil moisture model were collected in 2016 at the ICOS ecosystem station in Hyltemossa, in central Skåne (ICOS 2016). Daily precipitation data were obtained from SMHI station Klippan which is the SMHI station closest to Hyltemossa. Air temperature data had to be obtained from SMHI station Hörby since no temperature data is available from station Klippan. Accumulated rainfall in 2016 was 271 mm lower compared to 2015 resulting in dry conditions during May and June 2016 (Figures S1 and S2). For both years a decreasing *d* index with increasing soil depth is indicating an increasing disagreement between estimated and observed soil moisture values. In 2015 a negative Bias for the upper soil layer (0-6 cm) was observed, indicating an underestimation of the actual soil moisture. In contrast, a positive Bias was observed for deeper soil layers, indicating an overestimation of the actual soil moisture. Although the overall model performance in 2015 was acceptable, a comparatively low *d* index during the generative growth phase is indicating a disagreement between estimated and observed soil moisture during this growth phase. In 2016 a general tendency for soil moisture overestimation was found. In both years a tendency for underestimation of the actual soil moisture in the first half of the year as well as an overestimation for the second half of the year was observed (Fig S3 and S4).

With an average *d* index of 0.698 over both tested years, the overall performance of the soil moisture model was acceptable, although during the generative growth phase in 2015 the prediction accuracy was poor. A previous validation of the model in southern Quebec, Canada, described a decreasing prediction accuracy with increasing soil depths which is in accordance with our observations. In addition we can derive from our validation that the model accuracy seems to be higher in years with relative water shortage i.e. when seeds tend to be less dormant. The STM^2^ soil moisture model does not take into account a soil depth dependent decrease of organic matter, rather than a constant soil organic matter content had to be assumed. Soil organic matter content is positively correlated with the available water capacity. An increase in organic matter from 0.5 to 3% can result in a more than doubled available soil water content (Hudson 1994). Since usually soil organic matter content is decreasing with increasing soil depth, one must assume the same for the available soil water content. The observed tendency for overestimation of soil moisture in deeper soil layers as well as the observed increasing deviation between estimated and observed values with increasing soil depth might partly stem from this shortcoming. As pointed out above, *A. myosuroides* is building only a shallow rooting system, as suggested by the fact that only the top 10 cm soil layer appeared to be relevant for water stress. Thus, we can accept this limitation of the model. However, for weed species with deeper going root systems this might become a relevant drawback.

FIGURES

**Figure S1** Climate diagram for Klippan/Hyltemossa 2015. Red line; monthly cycle of mean temperature in °C. Blue line; monthly cycle of precipitation sum in mm. Red dots are indicating arid conditions. Daily maximum average temperature of the hottest month and daily minimum average temperature of the coldest month labelled in black at the left margin of the diagram. Annual average temperature and precipitation sum are given in black at the upper margin of the diagram. Light blue colour at the x axis is indicating months with probability for frost. Dark blue colour at the x axis is indicating months with sure frost. Temperature data from from SMHI station Hörby, precipitation data from SMHI station Klippan.

**Figure S2** Climate diagram for Klippan/Hyltemossa 2016. Red line; monthly cycle of mean temperature in °C. Blue line; monthly cycle of precipitation sum in mm. Red dots are indicating arid conditions. Daily maximum average temperature of the hottest month and daily minimum average temperature of the coldest month labelled in black at the left margin of the diagram. Annual average temperature and precipitation sum are given in black at the upper margin of the diagram. Light blue colour at the x axis is indicating months with probability for frost. Dark blue colour at the x axis is indicating months with sure frost. Temperature data from from SMHI station Hörby, precipitation data from SMHI station Klippan.

**Figure S3** Top: air temperature (red) and daily precipitation sum (blue) for 2015, Klippan/Hyltemossa site. Bottom course of measured soil moisture (dotted line) and modelled soil moisture (solid line).

**Figure S4** Top: air temperature (red) and daily precipitation sum (blue) for 2016, Klippan/Hyltemossa site. Bottom course of measured soil moisture (dotted line) and modelled soil moisture (solid line). Soil moisture data for day 300 onwards were not available for 2016 for the study site.

**Table S1** Sampling site description.

| Sampling site | Year | Proportion of dormant seeds (%) | Longitude | Latitude | Sand (%) | Silt (%) | Clay (%) | Nearest SMHI weather station | Distance to weather station (km) | Start generative phase (day±7) | 10-30% seed shed reached (day±7) | Precipitation sum during generative growth phase (mm) | Temperature sum during generative growth phase (°C) | Days with mean ψ < FC during generative phase (2-10 cm soil depth) | Days with mean ψ < PWP during generative phase (2-10 cm soil depth) |
| --- | --- | --- | --- | --- | --- | --- | --- | --- | --- | --- | --- | --- | --- | --- | --- |
| Alberta | 2015 | 69 | 13.32 | 55.63 | 48 | 30 | 21 | Lund | 10 | 141 | 196 | 78 | 852.8 | 32 | 13 |
| Brönnestad | 2015 | 79 | 13.70 | 56.08 | 58 | 27 | 14 | Hörby A | 24 | 146 | 201 | 105 | 815.7 | 27 | 5 |
| Gylle | 2015 | 86 | 13.20 | 55.41 | 50 | 30 | 20 | Sturup | 17 | 147 | 202 | 109 | 817.1 | 24 | 8 |
| Jordberga | 2015 | 79 | 13.41 | 55.42 | 47 | 32 | 21 | Sturup | 12 | 146 | 201 | 108 | 810.5 | 24 | 8 |
| Klagstorp | 2015 | 79 | 13.37 | 55.39 | 50 | 30 | 20 | Sturup | 15 | 146 | 201 | 108 | 810.5 | 25 | 8 |
| Lönhult | 2015 | 51 | 12.71 | 56.19 | 36 | 33 | 31 | Barkåkra | 15 | 139 | 194 | 142 | 831.0 | 56 | 3 |
| Mossheddinge | 2015 | 47 | 13.28 | 55.62 | 50 | 29 | 21 | Lund | 9 | 141 | 196 | 78 | 852.8 | 32 | 13 |
| Rosengren | 2015 | 62 | 13.33 | 55.61 | 45 | 33 | 22 | Lund | 11 | 141 | 196 | 78 | 852.8 | 32 | 11 |
| Svedberga | 2015 | 50 | 13.33 | 55.70 | 58 | 25 | 17 | Lund | 6 | 142 | 197 | 78 | 859.3 | 35 | 14 |
| Trelleborg | 2015 | 70 | 13.16 | 55.40 | 51 | 29 | 20 | Sturup | 20 | 147 | 202 | 109 | 817.1 | 25 | 8 |
| Vejbygården | 2015 | 84 | 12.82 | 56.32 | 46 | 32 | 22 | Barkåkra | 3 | 147 | 202 | 142 | 831.0 | 14 | 1 |
| Örup | 2015 | 62 | 13.30 | 55.62 | 46 | 30 | 23 | Lund | 10 | 141 | 196 | 78 | 852.8 | 32 | 13 |
| Esarp | 2016 | 51 | 13.33 | 55.61 | 45 | 33 | 22 | Sturup | 11 | 135 | 190 | 93 | 881.3 | 38 | 21 |
| Gunnarstorp | 2016 | 58 | 12.97 | 56.10 | 53 | 29 | 19 | Helsingborg | 15 | 134 | 189 | 152 | 925.8 | 40 | 23 |
| Heagård | 2016 | 37 | 12.78 | 56.70 | 49 | 33 | 18 | Halmstad | 9 | 128 | 183 | 62 | 934.3 | 47 | 26 |
| Jordberga | 2016 | 58 | 13.41 | 55.42 | 47 | 32 | 21 | Sturup | 12 | 137 | 192 | 116 | 896.3 | 37 | 24 |
| Klagstorp | 2016 | 64 | 13.37 | 55.39 | 50 | 30 | 20 | Sturup | 15 | 133 | 188 | 86 | 871.7 | 41 | 24 |
| Kongsmarken | 2016 | 61 | 13.31 | 55.60 | 52 | 27 | 21 | Sturup | 10 | 135 | 190 | 93 | 881.3 | 40 | 26 |
| Krapperup | 2016 | 52 | 12.53 | 56.26 | 55 | 30 | 15 | Barkåkra | 20 | 134 | 189 | 96 | 910.4 | 39 | 24 |
| Lydinge | 2016 | 31 | 12.87 | 56.10 | 56 | 25 | 19 | Helsingborg | 10 | 128 | 183 | 96 | 929.9 | 47 | 29 |
| Ormastorp | 2016 | 59 | 12.87 | 56.13 | 52 | 26 | 22 | Helsingborg | 13 | 138 | 193 | 184 | 954.1 | 37 | 27 |
| Ullriksfält | 2016 | 69 | 12.86 | 56.25 | 60 | 21 | 18 | Barkåkra | 6 | 134 | 189 | 96 | 910.4 | 39 | 26 |
| Viarp | 2016 | 65 | 12.88 | 55.96 | 56 | 26 | 19 | Helsingborg | 10 | 134 | 189 | 152 | 925.8 | 41 | 25 |

**Table S2** STM^2^ model parametrisation

| Variable | Settings |
| --- | --- |
| Soil texture | Site specific, see Table S1 |
| Soil organic matter | 4% for all sampling sites |
| Weather data | Site specific, see Table S1 |
| Climate | Warm temperate rainy |
| Average wind speeds | 6 - 11 km/h |
| Soil properties | Default parameters calculated by the model based on soil texture and OM |
| Depth of the modelled soil profile | 1.5 m |
| Model boundary conditions temperature | Solar heating enabled with 75% shade |
| Model boundary conditions moisture | Evapration enabled as well as precipitation carryover with 50% runoff |
| Lower boundary condition | -500 kPa |
| Initial water content | -12 kPa |

**Table S3** Model performance measures d index, RMSE and Bias for evaluation of STM^2^ soil moisture model.

| Year | Soil depth | Julian day 0 - 365 | | | Generative growth phase (Julian day 120 - 220) | | |
| --- | --- | --- | --- | --- | --- | --- | --- |
|  |  | d index | RMSE | Bias | d index | RMSE | Bias |
|  | cm |  |  |  |  |  |  |
| 2015 | 0-6 | 0.770 | 0.057 | -0.026 | 0.338 | 0.083 | -0.070 |
|  | 5 | 0.730 | 0.058 | -0.027 | 0.221 | 0.081 | -0.068 |
|  | 10 | 0.457 | 0.084 | 0.076 | 0.214 | 0.060 | 0.050 |
|  | 30 | 0.591 | 0.067 | 0.060 | 0.335 | 0.042 | 0.035 |
| 2016 | 0-6 | 0.889 | 0.047 | 0.004 | 0.768 | 0.055 | 0.000 |
|  | 5 | 0.851 | 0.056 | 0.017 | 0.810 | 0.052 | 0.000 |
|  | 10 | 0.597 | 0.096 | 0.090 | 0.545 | 0.082 | 0.075 |
|  | 30 | 0.702 | 0.068 | 0.054 | 0.653 | 0.058 | 0.050 |

**Table S4** ΔAIC_c_, p-values and linear model R^2^ for different time frames, for the explanatory variables ΣT_i_, temperature sum and ΣP_i_, precipitation sum.

| Explanatory variable | Time frame (dbh) | AIC_c_ | ΔAIC_c_ | p value | Adjusted R^2^ |
| --- | --- | --- | --- | --- | --- |
| Temperature sum (°C) | 0-7 | 183.14 | 0.00 | 0.002 | 0.345 |
|  | 0-14 | 183.25 | 0.12 | 0.002 | 0.342 |
|  | 0-21 | 193.91 | 10.78 | 0.884 | 0.000 |
|  | 0-28 | 193.73 | 10.60 | 0.669 | 0.000 |
|  | 0-35 | 183.42 | 0.29 | 0.002 | 0.337 |
|  | 0-42 | 184.06 | 0.92 | 0.003 | 0.318 |
|  | 0-49 | 189.46 | 6.32 | 0.046 | 0.138 |
|  | 0-56 | 187.54 | 4.40 | 0.017 | 0.207 |
|  | 14-28 | 183.44 | 0.31 | 0.002 | 0.336 |
|  | 28-42 | 184.25 | 1.11 | 0.003 | 0.313 |
|  | 42-56 | 192.57 | 9.43 | 0.269 | 0.000 |
| Precipitation sum (mm) | 0-7 | 192.81 | 10.19 | 0.317 | 0.000 |
|  | 0-14 | 193.88 | 11.25 | 0.820 | 0.000 |
|  | 0-21 | 193.94 | 11.31 | 0.989 | 0.000 |
|  | 0-28 | 193.91 | 11.28 | 0.866 | 0.000 |
|  | 0-35 | 192.81 | 10.19 | 0.317 | 0.000 |
|  | 0-42 | 192.60 | 9.97 | 0.274 | 0.000 |
|  | 0-49 | 193.05 | 10.43 | 0.374 | 0.000 |
|  | 0-56 | 190.55 | 7.93 | 0.082 | 0.000 |
|  | 14-28 | 193.68 | 11.06 | 0.633 | 0.000 |
|  | 28-42 | 182.63 | 0.00 | 0.001 | 0.359 |
|  | 42-56 | 191.65 | 9.03 | 0.154 | 0.000 |

**Table S5** ΔAICc values for different time frames and soil layers for the explanatory variable ${\Sigma\psi}_{s,k}$. Time frame is given in days before 10-30% seed shed.

| Time frame (*s*) | Soil layer (*k*) | | | | | |
| --- | --- | --- | --- | --- | --- | --- |
|  | 2-10 cm | 2-20 cm | 2-30 cm | 10-20 cm | 10-30 cm | 20-30 cm |
| 0-7 | 14.31 | 14.26 | 13.75 | 11.60 | 10.33 | 11.74 |
| 0-14 | 10.15 | 9.22 | 7.99 | 6.89 | 6.23 | 13.79 |
| 0-21 | 11.55 | 11.63 | 10.70 | 7.98 | 7.88 | 12.85 |
| 0-28 | 6.54 | 6.71 | 5.02 | 3.32 | 4.73 | 12.65 |
| 0-35 | 0.56 | 0.93 | 0.37 | 0.07 | 2.47 | 12.71 |
| 0-42 | 1.99 | 2.06 | 1.20 | 1.72 | 3.69 | 12.92 |
| 0-49 | 4.50 | 3.55 | 3.61 | 4.10 | 5.22 | 13.29 |
| 0-56 | 3.63 | 3.31 | 4.12 | 6.15 | 6.60 | 13.29 |
| 7-14 | 0.56 | 0.97 | 0.97 | 3.52 | 3.70 | 14.31 |
| 7-21 | 6.34 | 7.79 | 7.39 | 7.08 | 7.85 | 13.61 |
| 7-28 | 4.41 | 4.82 | 4.27 | 4.71 | 5.91 | 13.13 |
| 7-35 | **0.00** | 0.60 | 0.81 | 1.75 | 3.67 | 13.09 |
| 7-42 | 1.14 | 2.06 | 2.09 | 4.07 | 5.24 | 13.21 |
| 7-49 | 5.36 | 5.00 | 5.48 | 6.39 | 6.78 | 13.53 |
| 7-56 | 4.87 | 5.02 | 6.11 | 7.98 | 7.91 | 13.53 |
| 14-21 | 14.11 | 14.31 | 14.30 | 13.04 | 12.28 | 11.88 |
| 14-28 | 10.71 | 11.20 | 10.49 | 9.07 | 8.91 | 12.35 |
| 14-35 | 4.27 | 4.76 | 4.80 | 4.47 | 5.41 | 12.84 |
| 14-42 | 5.93 | 6.89 | 6.78 | 7.50 | 7.55 | 13.09 |
| 14-49 | 9.33 | 9.05 | 9.32 | 9.30 | 8.91 | 13.46 |
| 14-56 | 8.43 | 8.66 | 9.41 | 10.25 | 9.67 | 13.46 |
| 21-28 | 11.53 | 10.70 | 9.04 | 7.63 | 7.21 | 12.83 |
| 21-35 | 4.76 | 3.64 | 3.21 | 2.88 | 3.47 | 13.19 |
| 21-42 | 6.00 | 5.97 | 5.89 | 7.27 | 6.93 | 13.34 |
| 21-49 | 9.92 | 9.08 | 9.32 | 9.59 | 8.90 | 13.67 |
| 21-56 | 8.91 | 8.76 | 9.52 | 10.54 | 9.74 | 13.67 |
| 28-35 | 1.77 | 1.77 | 2.35 | 3.16 | 3.80 | 13.77 |
| 28-42 | 5.38 | 6.34 | 7.26 | 9.43 | 8.74 | 13.81 |
| 28-49 | 9.85 | 9.42 | 10.61 | 11.27 | 10.34 | 14.05 |
| 28-56 | 8.84 | 9.15 | 10.61 | 11.88 | 10.89 | 14.05 |
| 35-42 | 13.98 | 14.02 | 14.02 | 13.84 | 12.59 | 13.88 |
| 35-49 | 14.09 | 13.62 | 13.98 | 13.67 | 12.45 | 14.22 |
| 35-56 | 12.31 | 12.29 | 13.15 | 13.58 | 12.39 | 14.22 |
| 42-49 | 14.27 | 14.25 | 14.30 | 14.03 | 12.65 | 14.03 |
| 42-56 | 12.92 | 12.84 | 13.51 | 13.81 | 12.52 | 14.03 |
| 49-56 | 7.48 | 9.19 | 10.99 | 13.46 | 12.30 | 13.97 |
